# Fretting & friction induced fatigue failure: damage criterion of polytetrafluoroethylene

**DOI:** 10.1016/j.heliyon.2020.e04066

**Published:** 2020-06-05

**Authors:** Quazi Md. Zobaer Shah, Mohammad Asaduzzaman Chowdhury, Md. Arefin Kowser

**Affiliations:** a Maintenance Department, Healthcare Pharmaceuticals Limited (Previously, Roche-BD), Rajendrapur, Bangladesh; b Department of Mechanical Engineering, Dhaka University of Engineering and Technology, Gazipur 1707, Bangladesh; c Department of Materials & Metallurgical Engineering, Dhaka University of Engineering and Technology, Gazipur 1707, Bangladesh

**Keywords:** Biomedical engineering, Tribology, Fatigue life prediction, Biomedical devices, Mechanics, Polymers, Materials characterization, Fretting ring, Friction pad, Fractographical demonstration

## Abstract

Biomedical implants like the hip joint with cup work under continuous friction and wear phenomena where soft materials are suitable for the low coefficient of friction. As continuous, joints go under dynamic fatigue that should be accelerated by the fretting action generated from contact pairs and the inclination angle of the femur. In this research, the fatigue behavior of PTFE has been studied and compared under friction along with fretting action. A FE based Numerical model justified the experimental results. It showed that fretting and friction influence the fatigue life of PTFE by various angles. Fretting pressure optimization was identified as the determinant factor, while the loading point ratio was remarked as an effective parameter for both fretting and friction fatigue. Penetration depths proportionality to corresponding stress observed the effect of fretting fatigue where friction acts in different degrees depending on the geometry (collar/notch)-loading (friction) position. The fractographical demonstration revealed a relation between crack orientation and fretting action. Predefined loading action on test samples justified the singularity of fretting-friction fatigue characteristics on the damage mechanism of PTFE.

## 1 Introduction

**Figure 1:**
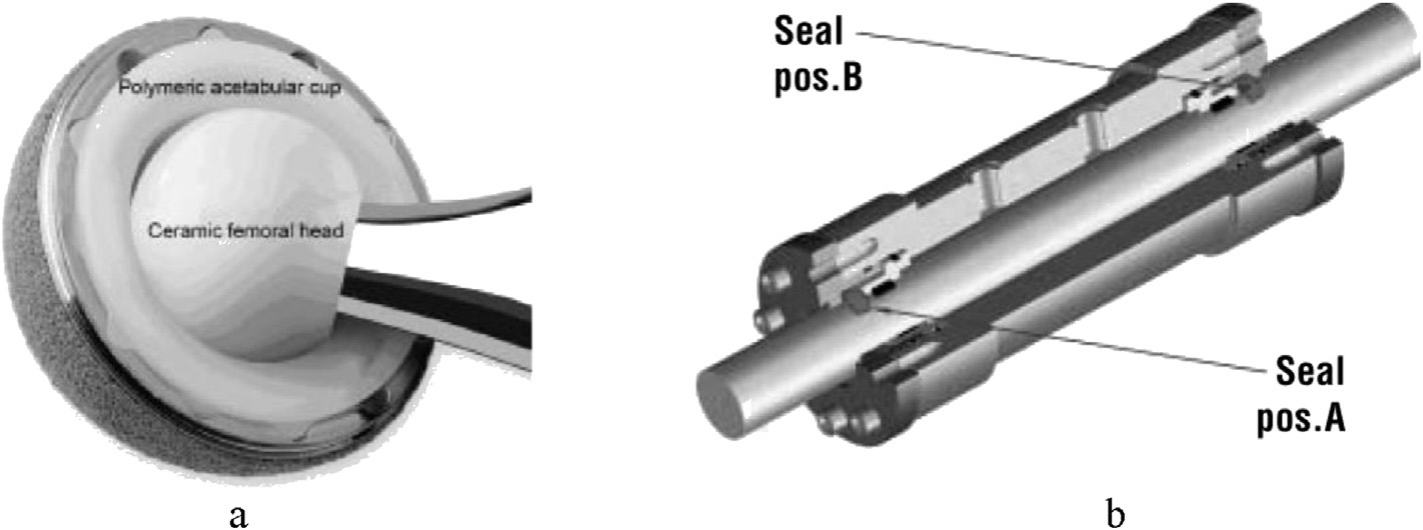
Hip joint cup (a) and Hydraulic cylinder pump (b) [24, 25].

In industrial applications, due to its low co-efficient of friction, PTFE (Polytetrafluoroethylene) is being used in the plain heading, gears, slide plates, seals, gaskets, bushings and more applications with the sliding activity of parts, where it works better than acetyl and nylon [1]. To resist fluid leakage from the cylinder when the system pressure pushes the piston inside the cylinder bore, PTFE piston with seals are desired. Rod seals prevent high-pressure fluids from passing out of the cylinder in a linear bi-directional motion. PTFE is preferred as a chemically neutral material of nuclear valves and seals. Easily machined, compounded PTFE elements are used in cases where elastomeric seals cannot sustain the temperature, frictional, or chemical resistive requirements of the application. Figure 1 represents the use of PTFE components for the hip joint cup, piston rod, seal and others. When friction acts along with fatigue, the stress, as well as the life cycle is influenced-thus fretting fatigue occurs [2] that can be classified into reciprocating and rotating type fatigue [3]. Along with recent researches on various ferrous and non-ferrous alloys, the fretting fatigue behavior of polymers can be found to some extent. Unal & Sen [4] showed that, as the applied load as well as the abrasion surface roughness increases, the wear rate also increases. Aderikha [5] experimented on the effect of adhesive activation of PTFE powder by sodium ammonia solution on the geometry and frictional properties of the sintered polymer were also studied. The tribological response of PTFE filled with various surface-treated CF was studied in [6]. Selectively irradiated PTFE surfaces tribology behavior was observed in [7]. Klinedinst found the interaction between PTFE and carbon black [8]. A new formula to customize CF-PES composite (carbon fabric-Polyethersulfone) with nanoparticles of PTFE was studied in [9]. The study in [10] showed the results of a experiment to quantify the effect of coated PTFE and deposited on a steel substrate. Lyutakov tested the electrical properties of flash-evaporated carbon nanolayers on PTFE [11]. FCP (fatigue crack propagation) angles for fatigue tests were studied in [12]. As a femoral shaft, clear Teflon was considered [13] where Teflon goes under some bending stress. As a prosthetic knee resurface implant [14], PTFE demonstrated effective biocompatibility for about 23 patients. Because of the low coefficient of friction, it shows excellence in biomedical contacts where fatigue strength under contact friction i.e. fretting fatigue of PTFE should be considered. Some little experiments were done on the tensile & creep behavior of Teflon polymer in [15, 16]. H. Aglan et al. [17] established an MCL (Modified Crack Layer) model to characterize FCP behavior and fracture resistance.

The tensile and compressive mechanical properties of PTFE were established in [18, 19]. They used a Hopkinson bar where a temperature series was undertaken for tension and compression tests. Failure behavior and microstructure were correlated to temperature-induced transition. It was found that along with crystallinity, mechanical properties of PTFE are influenced by strain rate and temperature. Current authors showed [20] the influence of sliding friction on the fatigue life of PTFE. If the action of friction is considered on the surface of PTFE, sliding and static friction can be observed. Moreover, in some negligible cases of consideration [21, 22] for varying angled eccentric force components other than the vertical and horizontal ones, bending occurs along with compression-tension. However, it should be considered with importance for PTFE materials, unlike ferrous alloys. Specifically, if friction occurs rigidly in any surface contact body under bending fatigue, it is termed as fretting fatigue. Surprisingly, there exists almost no information on the bending fretting fatigue behavior of PTFE.

In this present investigation, bending fatigue life, as well as the strength of PTFE, has been evaluated under the individual effect of fretting and frictional action. Experimental & numerical approaches were carried out. From available data, mutual relationships were observed for parameters like penetration depth, fatigue life, stress, etc. Physical fracture geometry demonstrated the effect of varying positions and action stresses. Later, it was compared with the previous research on friction fatigue that shows good agreement in the result of data analysis.

## 2 Methodology

### 2.1 Experimental approach

In this research work, a rotating bending fatigue testing machine was implemented where the stress-controlled cycle scheme was used. It consists of motor, frequency controller (VFD), cycles counting scheme, shaft coupling, and vertical load wheel stand. The top wheel is mounted on a screwed vertical shaft. When the wheel is rotated in the clockwise direction, it pulls up the shaft which is connected to a multi-axis rotating bearing that implies load on the end corner of the specimen. The head/Collar side of the specimen is adjusted and in alignment with the shaft coupling that rotates without any slipping of the surface body. The load wheel shaft was calibrated and along with it, a scale in Newton is vertically positioned that measures the forces applied on the specimen, by each turn of the top wheel. Once the force is measured, the stress can be obtained from the bending stress formula.

$$\sigma_{\mathrm{bending}}=\frac{\mathrm{My}}{I}$$

$$\text{Or,}\;\sigma_{\mathrm{bending}}=\frac{\mathrm{FL}}{\frac{\pi d^{3}}{32}}$$

here, d, L and F indicate the diameter of the specimen, body length, and load on the end, respectively.

**Figure 2:**
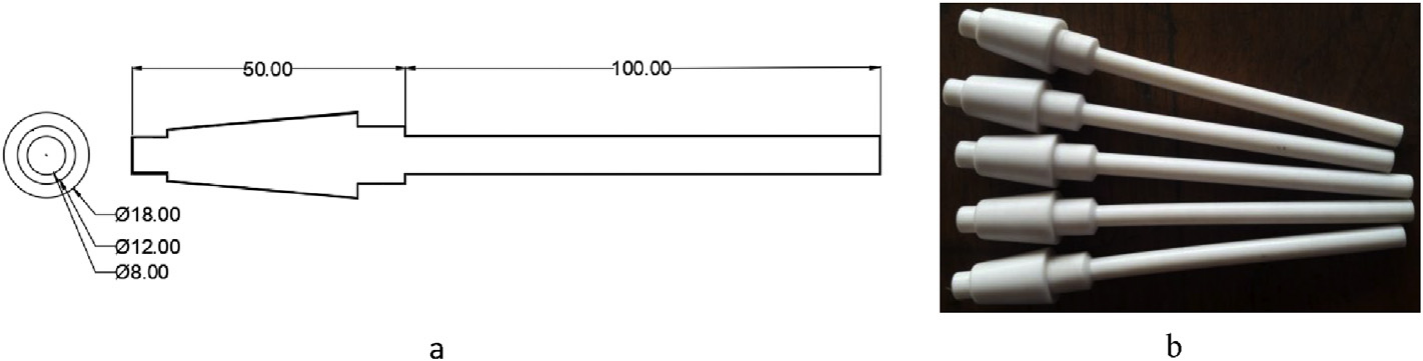
Fatigue test specimen: (a) Dimension (in mm) and (b) fabricated samples.

As the motor is switched on, it rotates the cylindrical specimen. Once, any load is implied on the specimen, it cracks at the collar/neck zone after a certain period, termed as Cycle, N. An S-N curve can be constructed from the availed stress and lifecycles value. On the failure and frequency setting of the specimen, the cut-off switch and the inverter are used to stop the machine, while the auto cycle counting system counts the cycles. Polytetrafluroethelyne (PTFE), a fluorocarbon solid was used as the polymer specimen. It is hydrophobic, has a low coefficient of friction (COF) and consists of only carbon and fluorine. Specimens were prepared from the Teflon bar as per sample design by a CNC lathe machine. As Teflon reacts to a higher temperature, all the tests were done at ambient temperature. For accuracy, a minimum of 3 specimens was considered ideal for each conditional test, if no irregularities were found in the result for a batch. Cylindrical cantilever specimen's properties and dimensions are given in Table 1 and Figure 2, respectively.

**Table 1:** Mechanical properties.

Material	Density kg/m^3^	E GPa	Poisson'sratio	YTS MPa	UTS MPa
Teflon (PTFE)	2300	0.5	0.46	30	43

**Figure 3:**
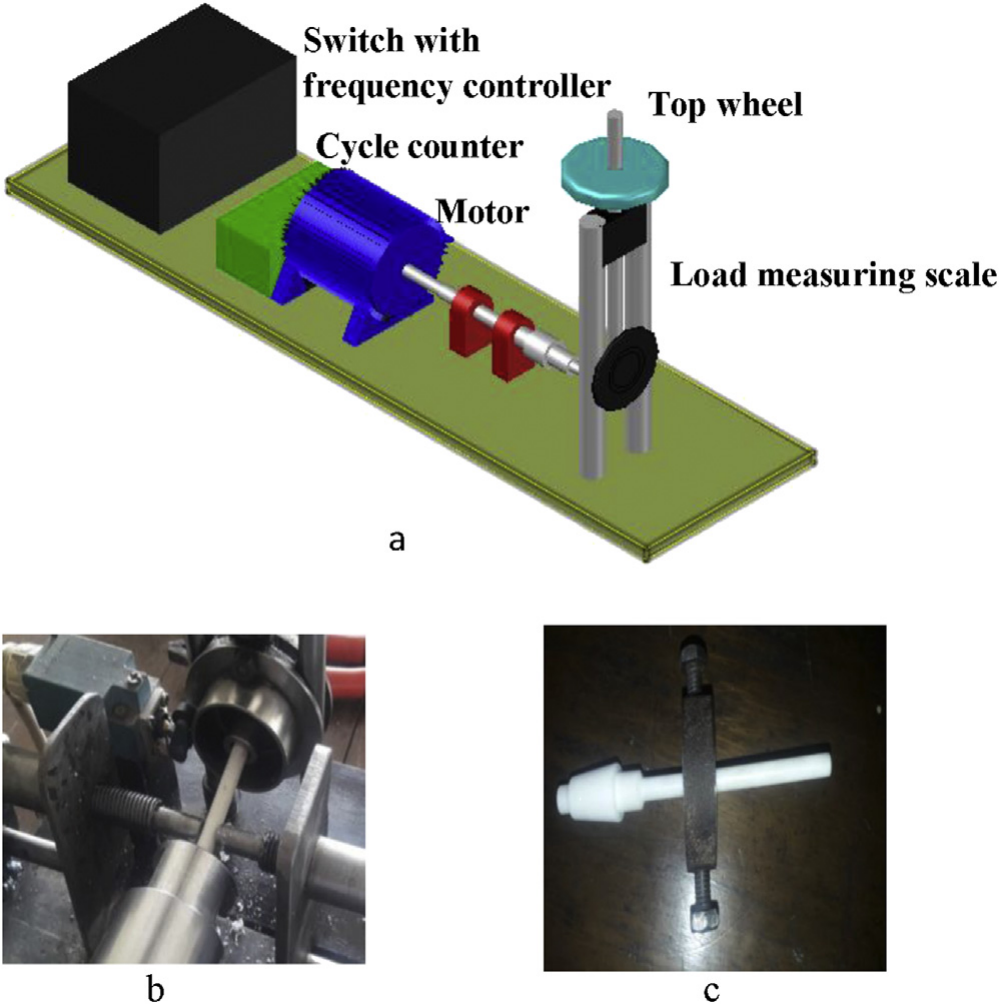
Rotating bending fatigue machine (a). Friction fatigue (b) and Fretting fatigue (c).

The Fretting phenomenon was confirmed and occurred at the surface area where fretting pads create pressure on the specimen. Along with the bending fatigue stress, fretting pressure is implied by the tightening screw. The fretting proving ring was located between 10 to 15 mm from the collar of the specimen. The fretting pressure influences the effect of bending stress that accumulates at the neck of the specimen. The flat to cylinder connections are confirmed by cylindrical fretting pads. Stiffer materials are appropriate for fretting pads than the test sample. Mild Steel bolts of 8 mm diameter were used as the fretting pads. The loading of the fretting is provided by a rotating circular proving ring. This ring system with inserted screwed bolts is given as shown in Figure 3 (c, below right) for the use of fretting fatigue. Adjustment of the lead screw by torque driver through the support ring meant the contact load.

The load cell measures the contact load while the friction testing machine can calculate the friction force. Such experimental procedure details can be found elsewhere [23]. Figure 3(a, upper) shows the experiment setup. As shown in Figure 3(b, below left), a screwed friction pad pair employed with a reduction gear system adjusts auto contact friction and controls the predefined sliding speed of the pads.

### 2.2 FEM approach

**Figure 4:**
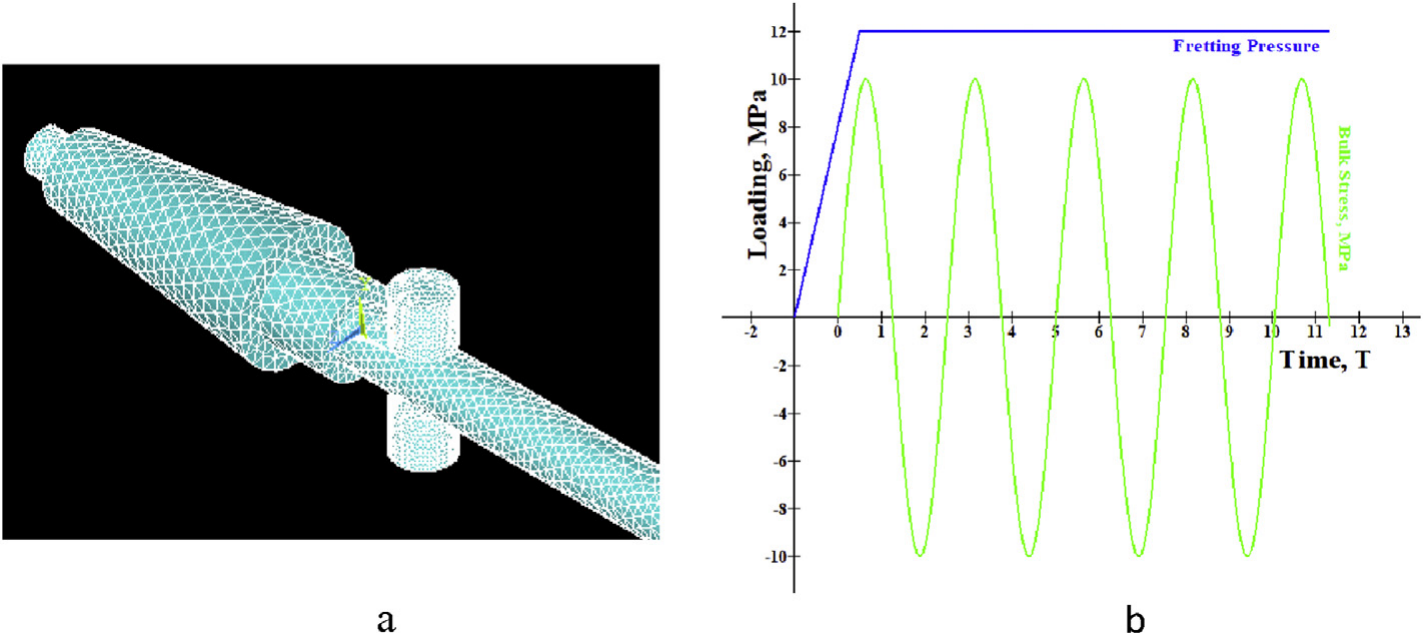
Constructed Geometry with mesh convergence (a) and loading sequence (b).

**Figure 5:**
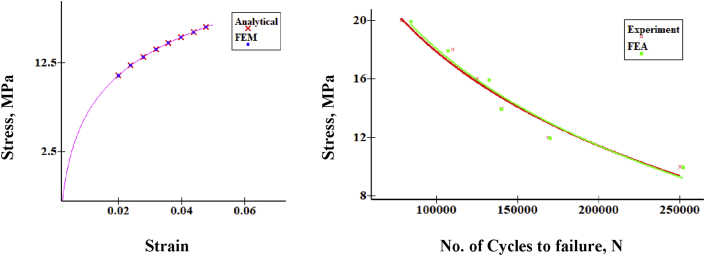
Graphical Comparison for FEA results with analytical and Experiment results.

For finite element assessment, ANSYS 17 was used here as an FE tool. CONTA 174 and TARGE 170 were selected as contact and target components, respectively. To converge the results, Adaptive mesh refinement was adopted. Since coarse mesh yields inaccurate results and extremely refined mesh consumes CPU runtime, the refinement process was continued until the reasonable convergence for the stress level was observed. It was found as convenient to refine until two consecutive stress value difference lies within a considerable range. As ideal, convergence study was considered as acceptable when stress difference reaches about 2%. Gradual mesh convergence yields the element size independent result. The displacement of the fretting pads was set as Ux = Uz = 0, Uy = free. Selected proximity and curvature with elevated smoothness, the center of fine significance, and angle of the span were considered. Frictional contact pair was adopted to touch where originally there was zero penetration and gap. Augmented Lagrange formulation was used under the contact target's asymmetric conduct. Contact paths were created along the non-filleted edged corner of the specimen. Specimen conical head was regarded to have fixed support while at the end of the cylindrical tail the bending load was implied. Flat on cylinder type contact was used where the fretting pad radius was 4 mm. In the beginning, a small displacement was applied to the pads, then it was removed to determine whether or not any problem of singularity occurred. Two load steps were considered for bulk and fretting stress implementation. As a first step, the fretting load/pressure was applied to the top area of the fretting pads from zero to maximum pressure, which remains constant until the end of the experiment. A bending load, as well as stress, were implemented in the second step. However, unlike the fretting one, strain and strain are simultaneously imposed in a sinusoidal shape on the opposite sides of the cantilever tail samples. Figure 4 shows the typical condition of loading. Blue and red lines, respectively, indicate fretting and bending loads. For verification and validation purposes, stress-strain and stress-cycle curves show good agreement between experimental and FE model either in ABAQUS [26] or in ANSYS [23, 28]. Analytical and experimental methods use conventional equations and formulas (Stress, σ = E. ε, (ε = Strain) and Stress, S = AN^B^, (N = cycles)) irrespective of the elements while FEA calculates results that consider the elements of geometry. As shown in Figure 5, FEM findings are in excellent agreement with the analytical and experimental results.

## 3 Results & discussion

It was found that both the friction and fretting cause failure in the same way-based on the fretting/ friction action point. In this experiment, this type loading cut-off point was about 70 mm from the free end i.e. fatigue characteristics and location alters as the fretting/friction point deviates at the 70 mm distance from the free end.

### 3.1 Fretting fatigue

**Case** **1**: Fretting acting above the loading cut-off point (a point above 70 mm) from the tail side.

**Figure 6:**
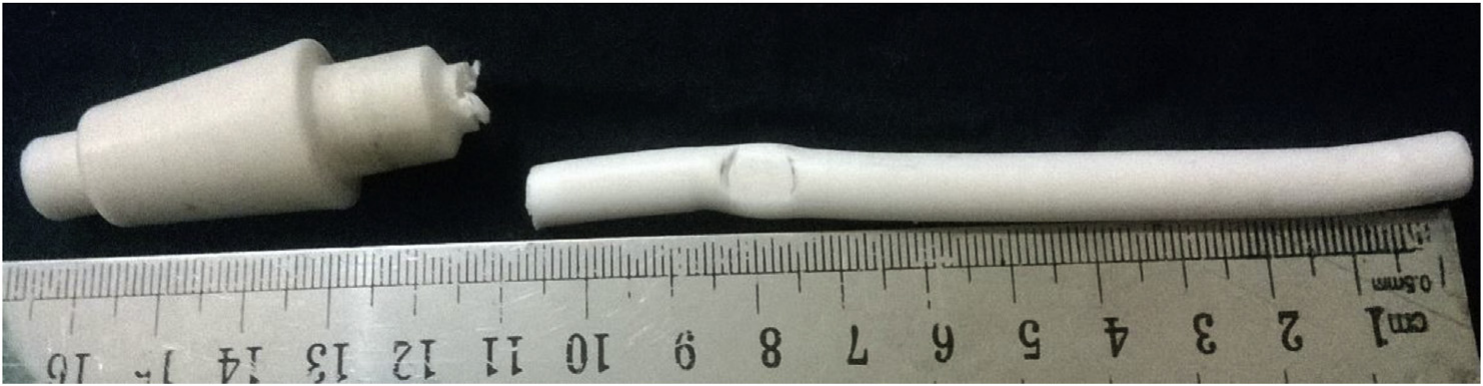
Fractured PTFE specimen under loading at a point above 70 mm from the tail side.

From Figure 6, it is found that, fracture occurred near the curvature/a point where smallest diameter meets the greater one. This may be because of the dual effect of edge and fretting action.

**Figure 7:**
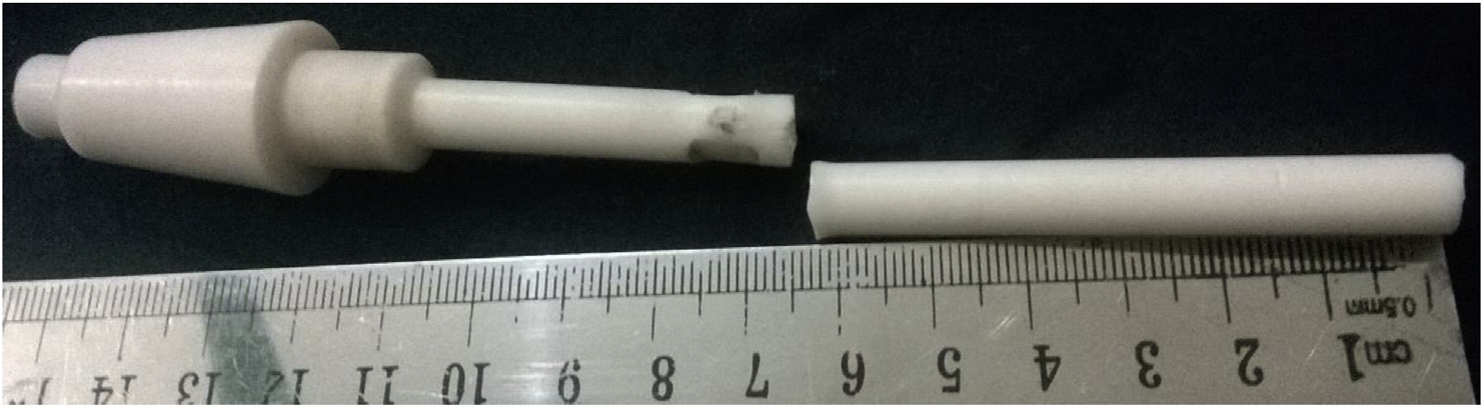
Fractured PTFE specimen under loading at a point below 70 mm from the tail side.

**Case 2**: Fretting acting below the loading cut-off point (a point below 70 mm) from the tail side.

**Figure 8:**
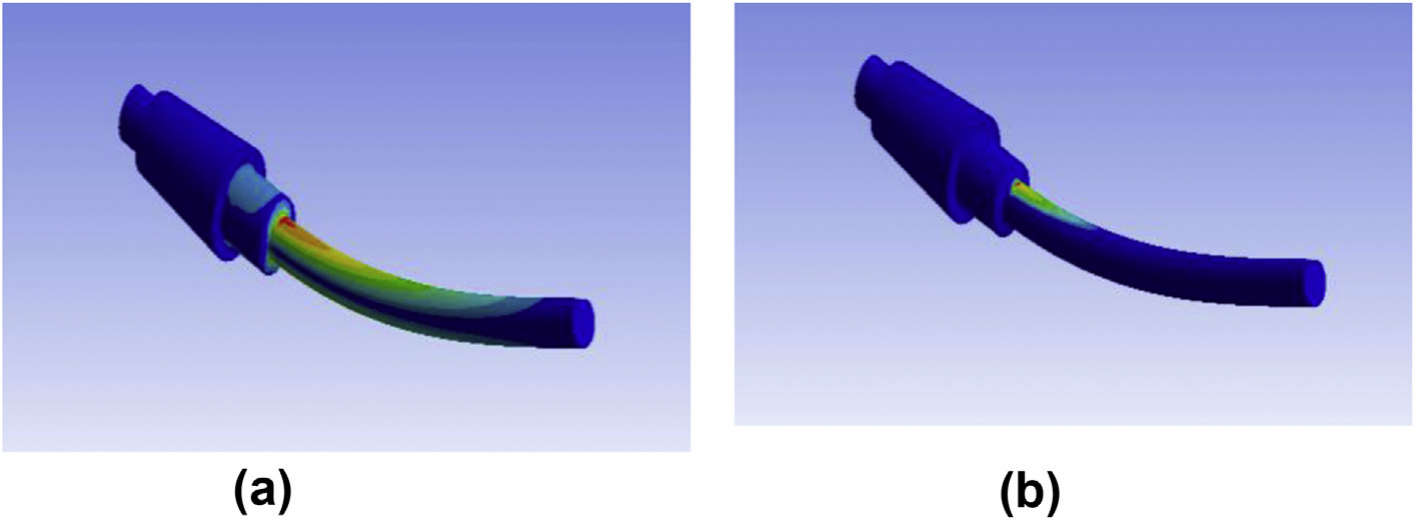
(a) Maximum stress and (b) Fatigue life at 8 N bending load for PTFE.

**Figure 9:**
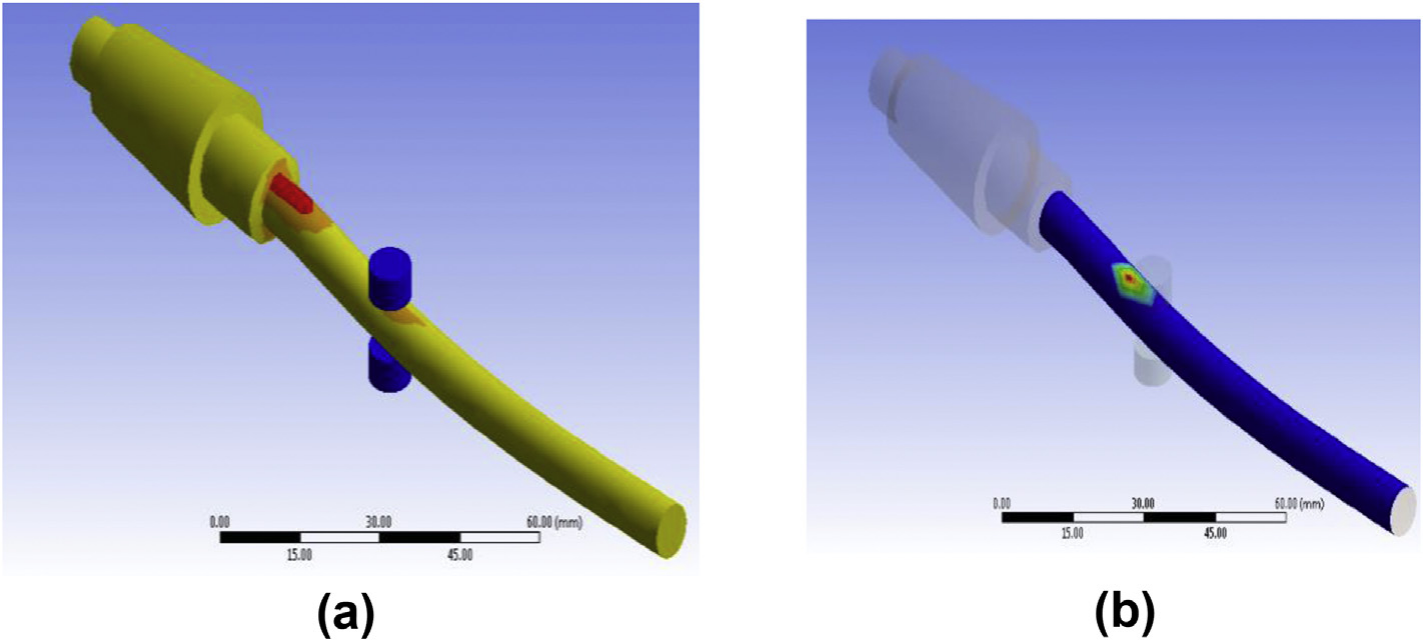
(a) fatigue life and (b) penetration at 8N bending load for PTFE.

Sample cracked just near the fretted zone (see Figure 7). It experiences comparatively more cycles before fracture at the same fretting load and jockey load under which loading at a point above 70 mm could not pass. Figures 8 and 9 shows contour outputs for 8 N loading. Blue zone and red zone shows the minimum and maximum contour parameter (i.e. stress/life), respectively. From this, it is obvious that, fretting accelerates the stress concentration as well as failure at the corner.

**Figure 10:**
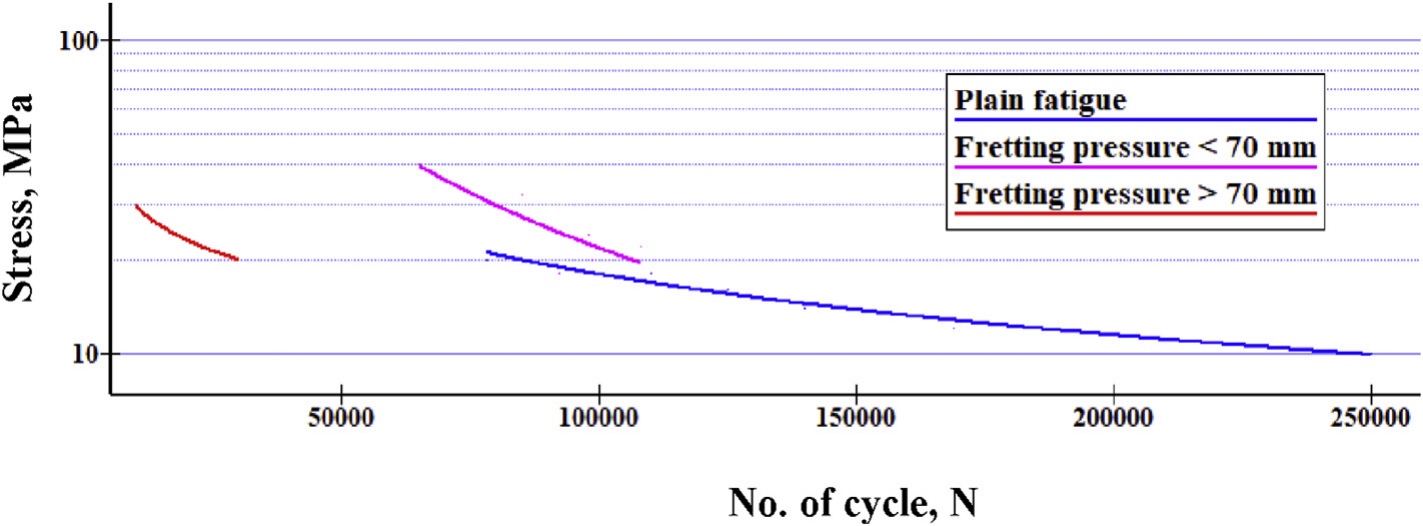
Fatigue-life comparison for normal and fretting fatigue of PTFE.

From the S-N diagram as shown in Figure 10, it can be said that, fatigue life decreases gradually and tends to become horizontal to baseline axis.

**Figure 11:**
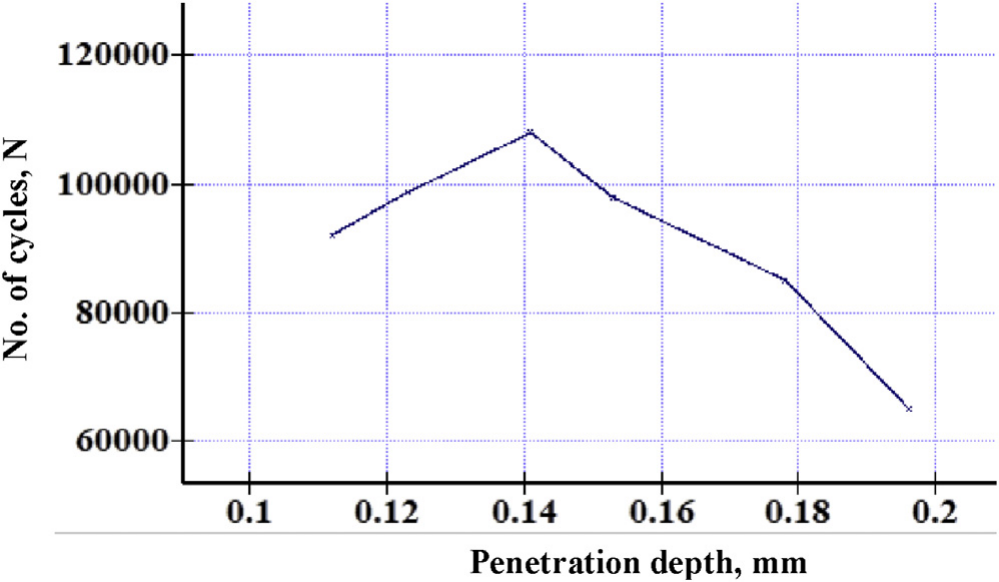
Fatigue life Vs. Penetration.

**Figure 12:**
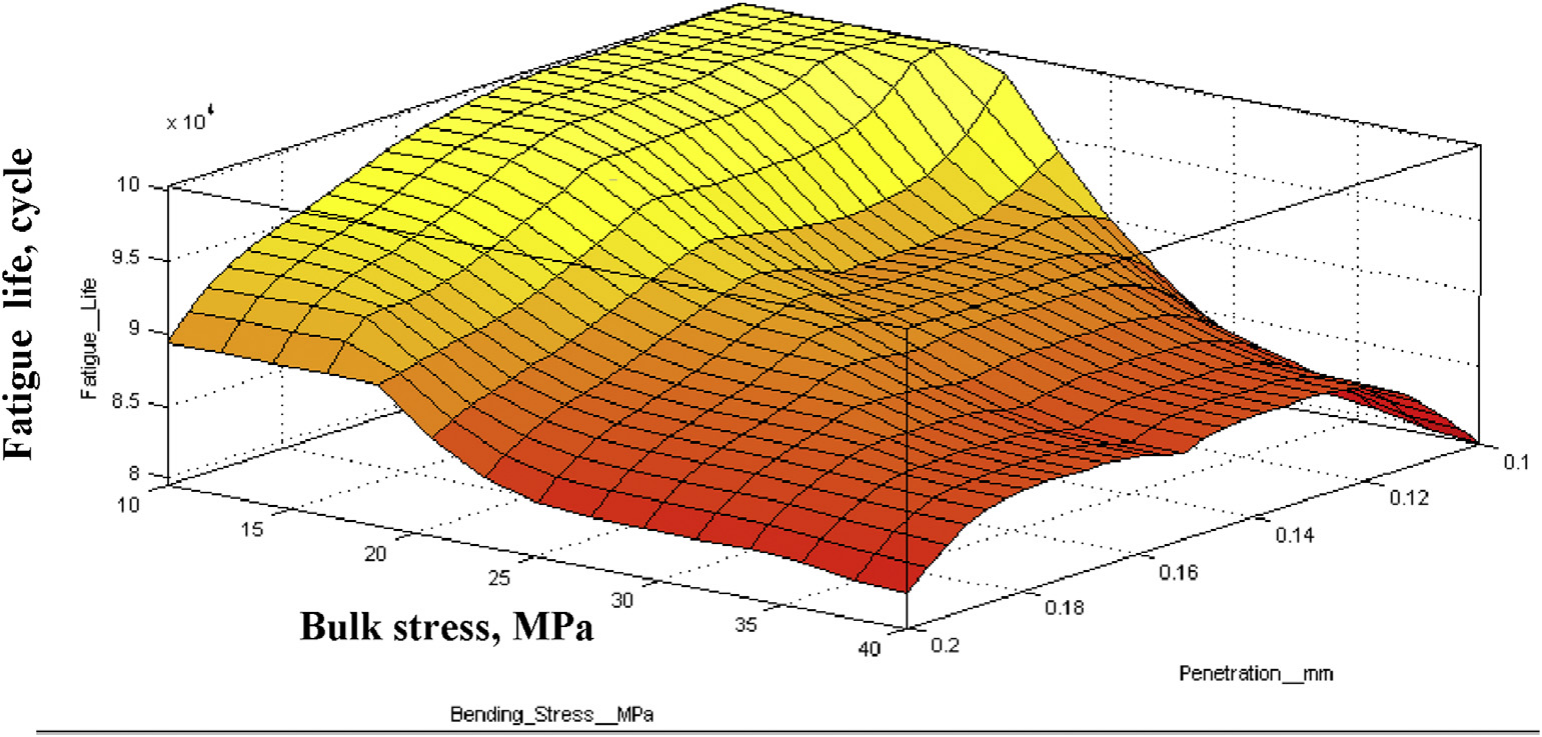
Relationship among bulk stress, penetration depth and fatigue life.

Comparing fretting fatigue with the normal one as shown in Figure 10 indicates that along with fretting, fatigue life-time line meets half the normal fatigue lifetime line. Fretting acting at above 70 mm from the free end affects badly than other cases. For with and without fretting, fatigue strength is 1.1558 × 10^−7^MPa and 0.2231 MPa at 10^7^ cycles respectively. Fatigue strength reduced by more than 90% because of fretting. As the penetration depth increases, fatigue life also increases up to a maximum life cycle. Figure 11 presents that because of constant fretting load on the contact pad, Teflon tends to bend as a polymer. When bending loads are gradually increased, it tends to straighten the cylinder and thereby tries to mitigate the fluctuation range that lessens fatigue life. However, after an optimized point, it just over necks the cross-section which could not assist the fatigue life cycle. It exists between 20-21 MPa bending stress at 0.14 mm penetrating depth and 108410 cycles, which shows a significant effect of penetration on fatigue life. Figure 12 shows the relationship between bulk stresses, penetration depth and fatigue life on a 3D surface graph.

**Figure 13:**
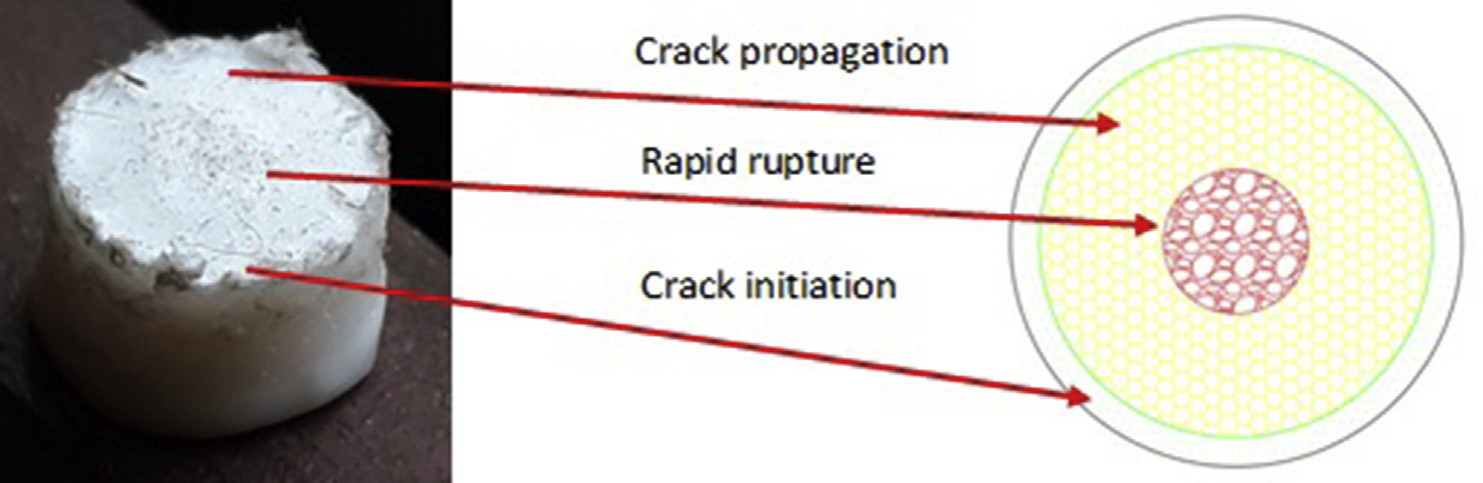
Normal fatigue.

**Figure 14:**
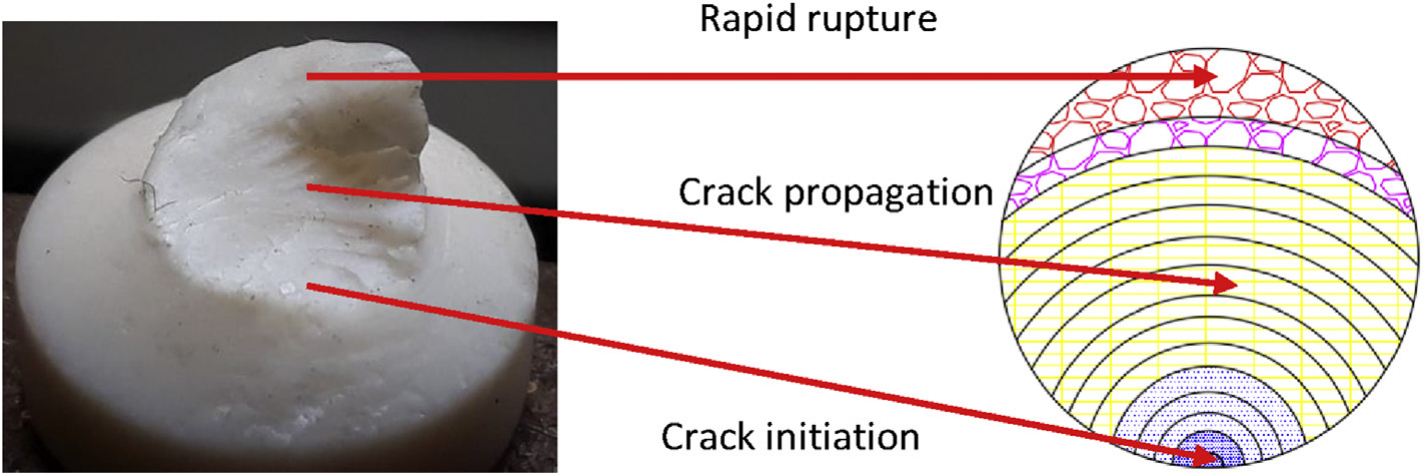
Fractured from fretting fatigue (acting above 70 mm from the tail side).

**Figure 15:**
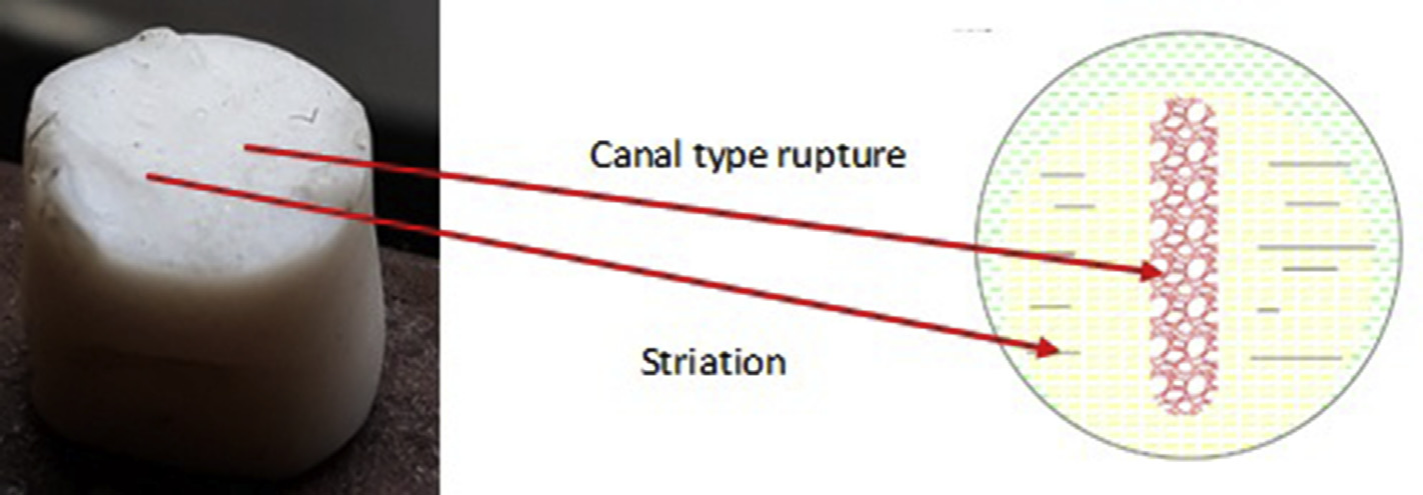
Fractured from fretting fatigue (acting below 70 mm from the tail side).

**Figure 16:**
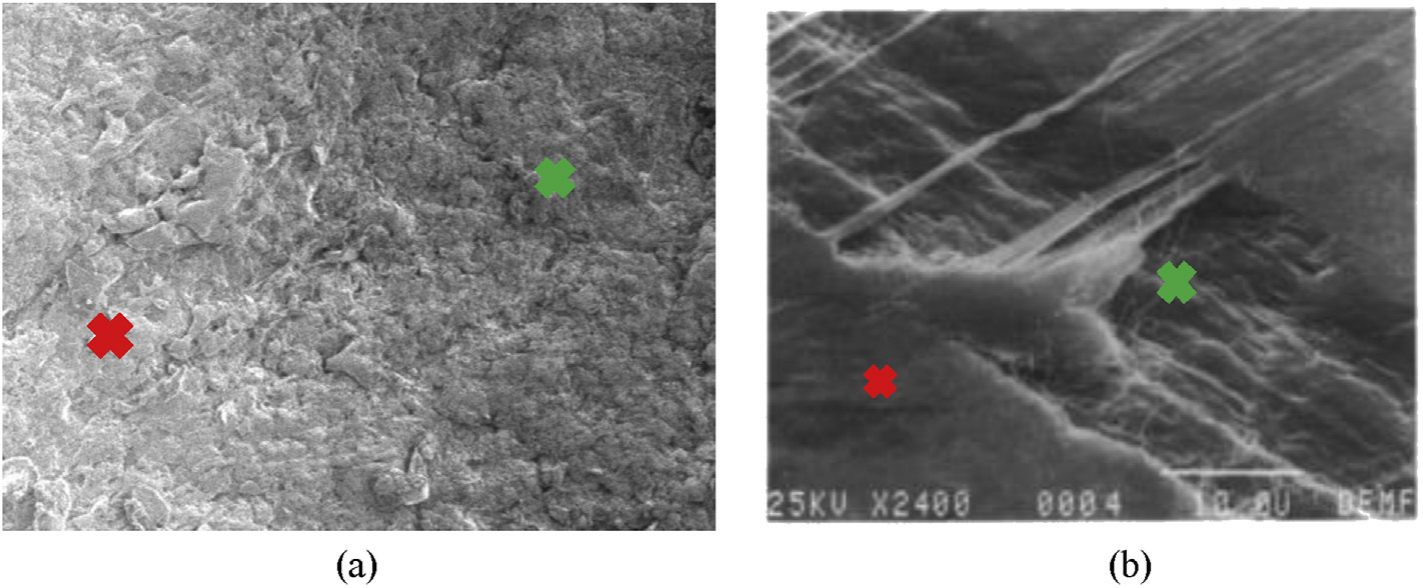
Electron image of (a) surface debris and (b) strip formation by sliding [27].

To show the clear distinguishable zones on the fractured surfaces of PTFE, schematic color drawings are demonstrated in Figures 13, 14, and 15. Unaided eye observation shows an almost circular shaped core which is comparatively rougher just like a nucleus (Figure 13). It is common in other ferrous alloys. When fretting is applied at above 70 mm from the free end of the sample, striation like a sound wave is formed gradually from a corner, called an initiation a point. Sudden & fast rupture is formed at opposite outside corners, in a circular crescent shape (Figure 14). The dual effect of the sharp edge (non-filleted) and the fretting caused the comparatively faster failure at the collar region. Interestingly, trench/canal type mid-core is found for fretting below 70 mm of the free end. Obvious striations along with canal type rupture are shown in Figure 15. Such striations, as well as benchmarks, are the results of stripped fibers of PTFE that forms due to the frictional movements. Near the trench/canal type fractured zone, along with the striations, a small amount of surface debris is also found as shown in Figure 16 (a). Figure 16 (b) shows the electron image of stripe formation by sliding wear [27]. Interestingly, trench/canal type mid-core is also found here under the sliding wear mechanism. Thus, the static friction (Fretting) and the sliding friction influenced the fractured surface almost in the same manner.

**Figure 17:**
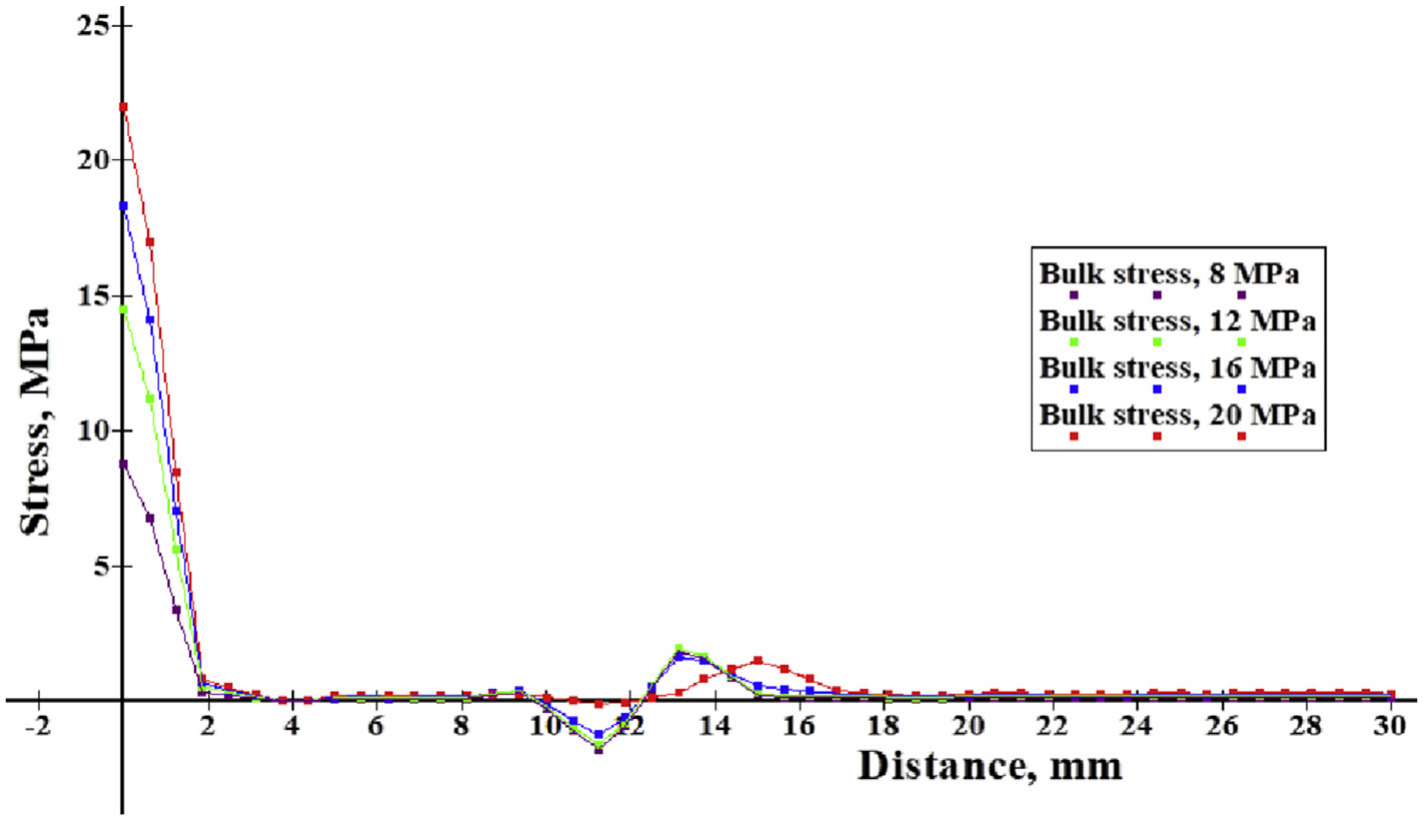
Stress distribution along flat on cylinder contact path for initial fretting load step.

**Figure 18:**
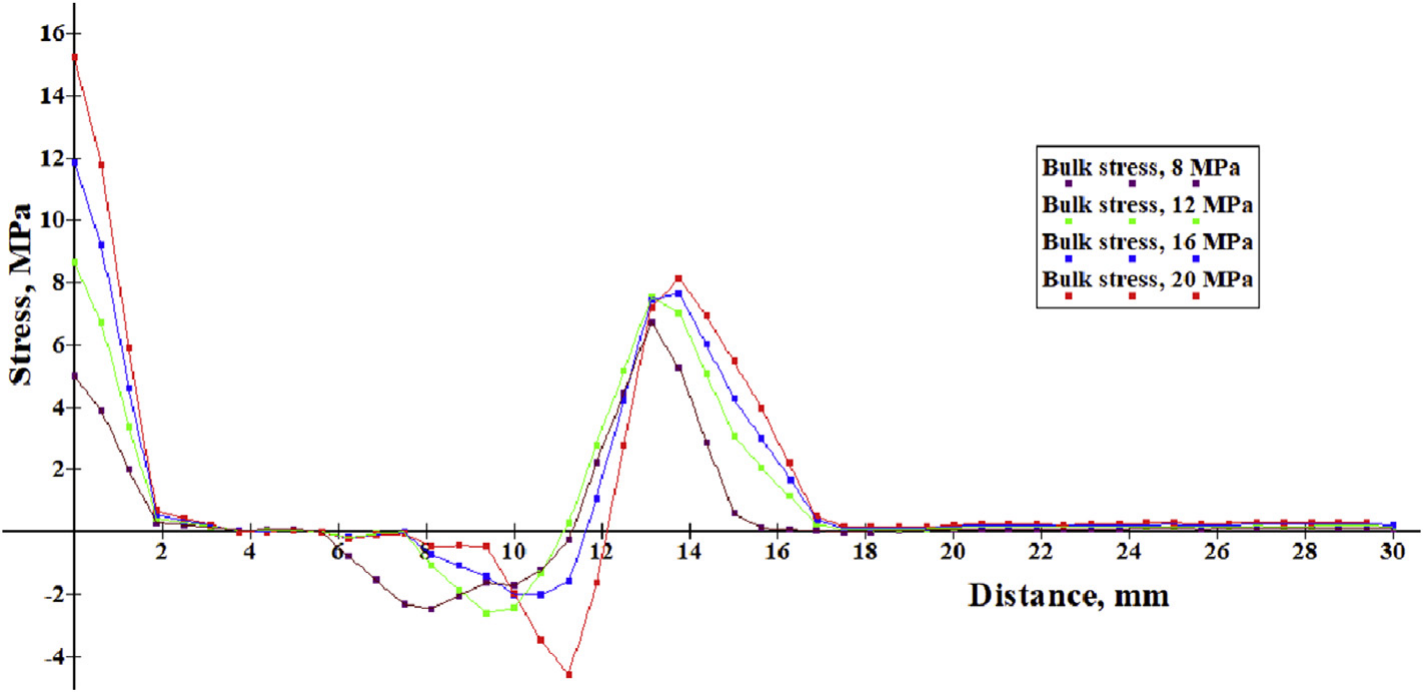
Stress distribution along flat-cylinder contact path for optimum fretting load step.

Finite element stress distribution along the contact path of friction pad and surface is shown in Figures 17 and 18. This justifies the fracture mechanism at the collar section of the specimen. At initial fretting step, higher stresses concentrate on the collar section that causes faster failure there. However, at the optimum fretting step as well as the penetration depth, significant stress crests are found at the fretting location while comparatively lower level stress gradients are found at the edge side. It seems like fretting sucks or pulls down the stress intensity from edge to contact area. As a result, fatigue life is developed for optimum penetration depth.

### 3.2 Friction fatigue

**Case 1**: Friction acting above the loading cut-off point (a point above 70 mm) from the tail side.

**Case 2**: Friction acting below the loading cut-off point (a point below 70 mm) from the tail side.

**Figure 19:**
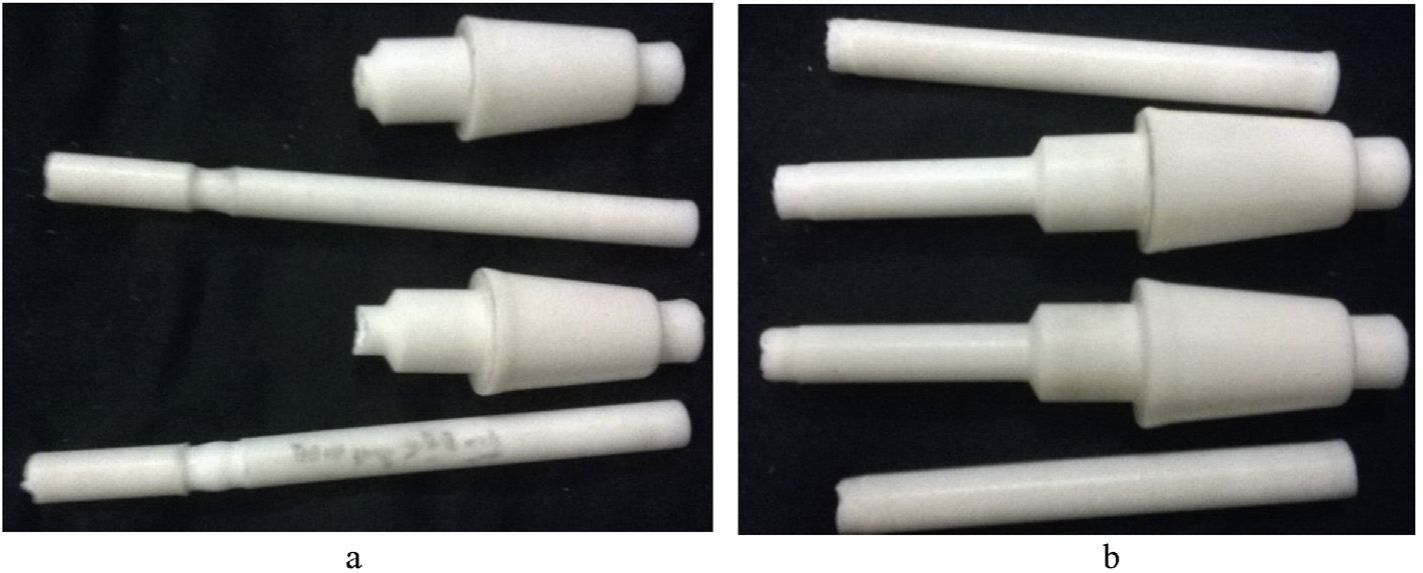
Cracked sample under friction a point of (a) above 70mm. (b) below 70 mm from the tail side [20].

**Figure 20:**
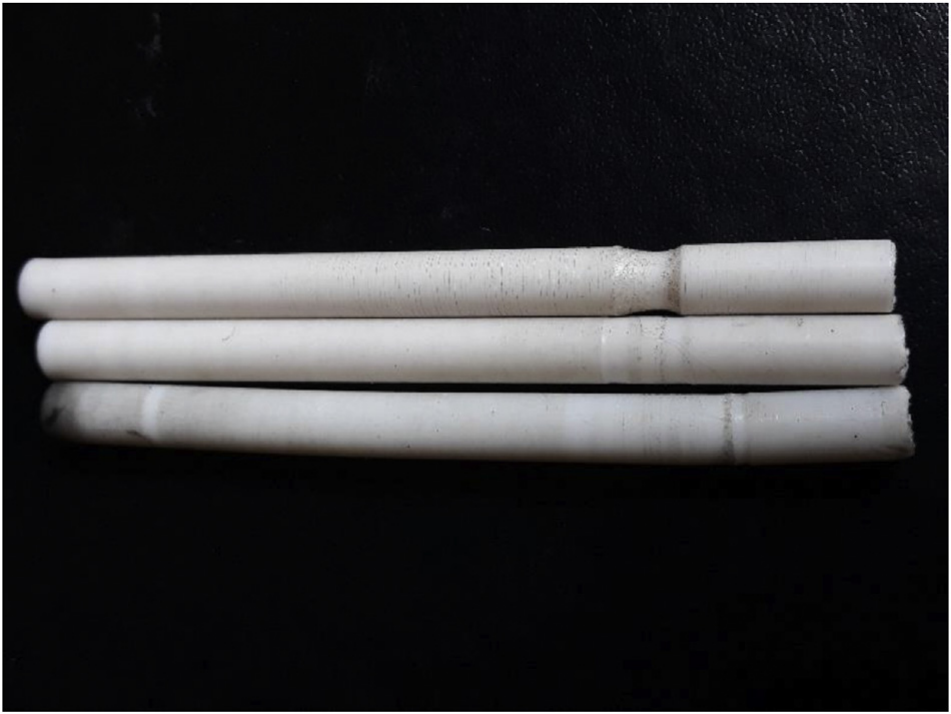
Cracked sample under friction a a point at above 70 mm from free end.

The same case happens for fretting fatigue (Figure 19). However, unlike fretting fatigue, it didn't show any optimum depth for friction wear, it cracked on the basis of loading a point even for a grooved sample (Figure 20).

**Figure 21:**
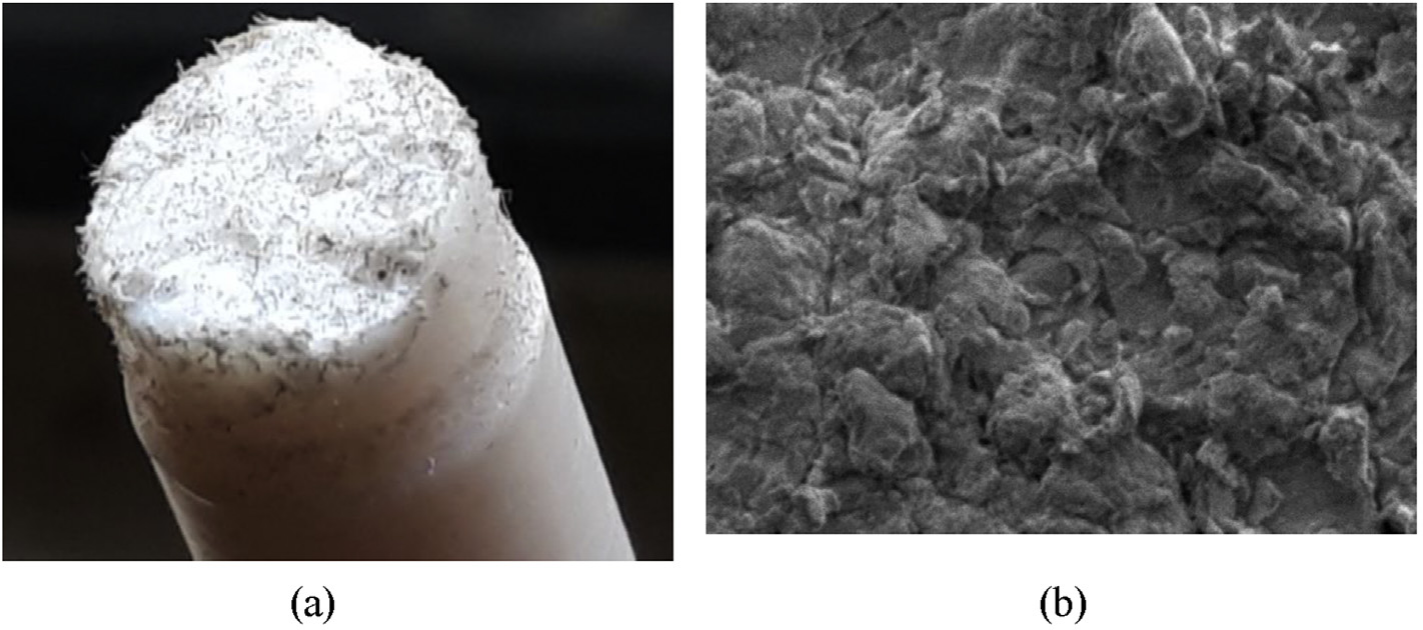
Friction fatigue,(a) Fractured surface,(b) Electron micograph.

Because of the friction fatigue, surface debris is formed as shown in Figure 21. Unlike the static (fretting) one, Sliding friction causes the uniform distribution of debris throughout the cracked zone, as well as the symmetrical view of the fractured surface. This cloud type debris is formed on the fractured surface much denser than the fretting fatigue case.

**Figure 22:**
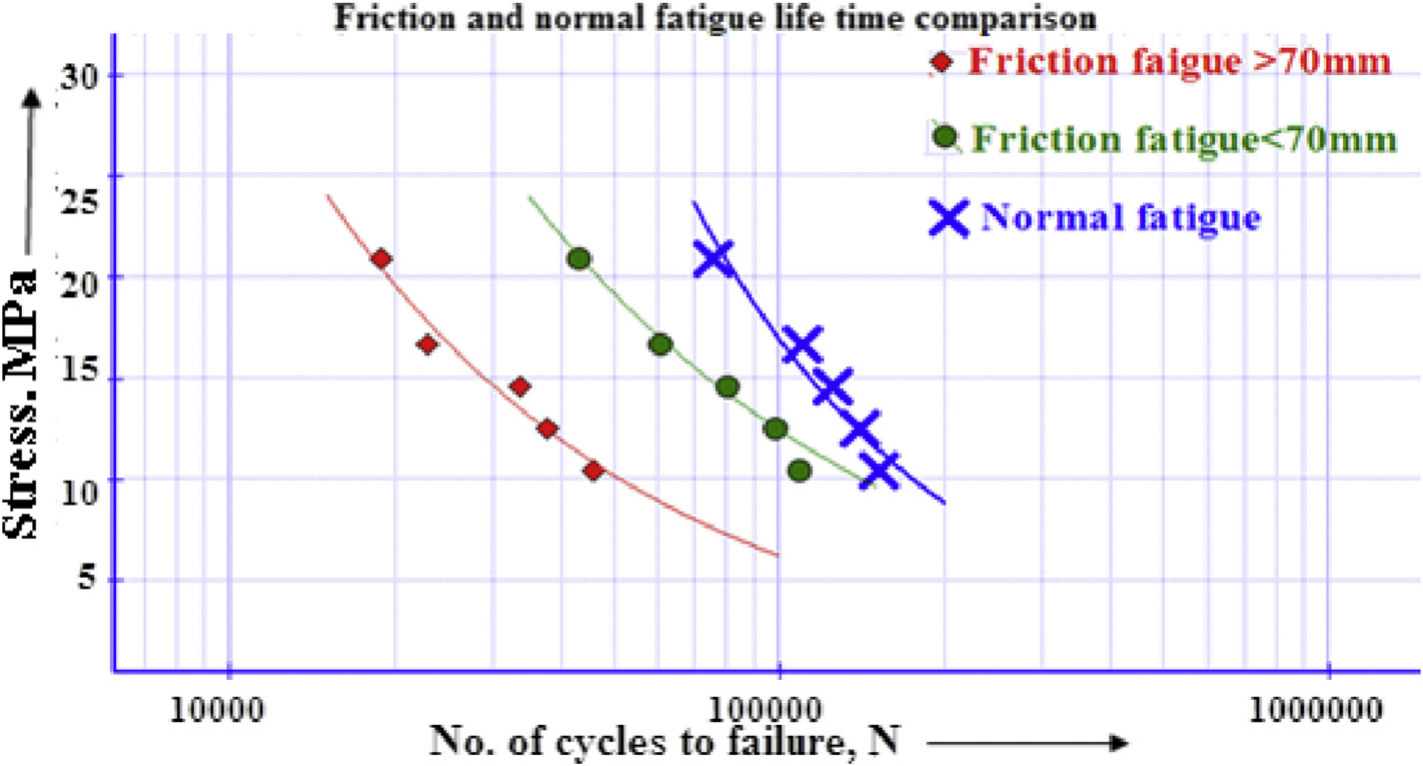
Comparison of S-N curves of PTFE [20].

Figure 22 shows the comparison of S-N curve for the same operating conditions. Fatigue life reduction percentage is greatly affected by pad position for friction. Because of the dual effect of collar & friction, it fails at the lowest cycles. However, it is not as distinguishable like as the case for fretting fatigue at above 70 mm from the end (Figure 10).

## 4 Conclusion

In this research, Rotating bending fatigue behavior of PTFE has been studied under the action of fretting and compared with the sliding friction effect. From the above mentioned two considerations, the outcome is summarized as followings:

- Reduction of fatigue life considers fretting & friction in two conditions. Within 70 mm from the tail side, fretting & friction causes fracture near the fretting ring and the fractioned zone, respectively. However, for the fretting/friction point above 70 mm, the combined action of fretting/friction with notch/collar causes it cracked near the collar side.
- Fretting fatigue reduces the lifetime of more than 90% for PTFE. Penetration depth increments up to an optimized point increases fatigue life because of balance in the constant negative contact pressure and positive bending loading. However, just after reaching that critical point, the fatigue life begins to decrease due to insufficient strength owing to tougher necking. Finite element stress distribution justifies the fracture at the collar as well as the development of the fatigue life by optimized the fretting pressure. However, for the friction fatigue, deeply grooved sample cracks at predefined a point.
- Fretting fractured surfaces demonstrated the importance of fretting action on failure mechanisms. Like other ferrous materials, it fails and cracks just like a dartboard section. For loading above 70 mm from the free end, it cracks and fractures in a crescent shape. Because of the dual effect of fretting and sharp edge geometry, faster failure occurs. The most important finding is that rapid rupture forms in a trench/ canal with striations shape that develops along with the action of loading, i.e., fretting pad position.

From the discussions above, it has been ended up with the observation of the fretting-friction effect on PTFE strength. This phenomenon should be considered while designing infrastructures associated with PTFE.

## Declarations

### Author contribution statement

Quazi Md. Zobaer Shah: Conceived and designed the experiments; Performed the experiments; Analyzed and interpreted the data; Wrote the paper.

Mohammad Asaduzzaman Chowdhury: Conceived and designed the experiments; Contributed reagents, materials, analysis tools or data.

Md. Arefin Kowser: Conceived and designed the experiments; Contributed reagents, materials, analysis tools or data.

### Funding statement

This research did not receive any specific grant from funding agencies in the public, commercial, or not-for-profit sectors.

### Competing interest statement

The authors declare no conflict of interest.

### Additional information

No additional information is available for this paper.

## References

[1] Munmaya Mishra, Yagci Yusuf (2016). Handbook of vinyl polymers: radical polymerization, process, and technology.

[2] Chowdhury M.A., Kowser M.A., Zobaer Shah Q.M., Das S. (2018). Characteristics and damage mechanisms of bending fretting fatigue of materials. Int. J. Damage Mech..

[3] Zobaer Shah Quazi Md, Chowdhury Md Asaduzzaman, Kowser Md Arefin (2019). On the diversity in design for different bending fretting fatigue mechanism. SN Appl. Sci..

[4] Unal H., Sen U., Mimaroglu A. (2007). Study of abrasive wear volume map for PTFE and PTFE Composites. Appl. Compos. Mater..

[5] Aderikha V.N., Novikov V.P., Filippovich S.R., Shapovalov V.A. (2018). The effect of PTFE powder adhesive activation on the wear resistance of block PTFE. J. Frict. Wear.

[6] Shi Yijun, Feng Xin, Wang Huaiyuan, Lu Xiaohua (2007). Tribological properties of PTFE composites filled with surface-treated carbon fiber. J. Mater. Sci..

[7] Blanchet Thierry A., Peng Yih-Lih, Nablo Sam V. (1998). Tribology of selectively irradiated PTFE surfaces. Tribol. Lett..

[8] Klinedinst K.A., Vogel W.M., Stonehart P. (1976). Rheological characterization and thermal degradation of PTFE. J. Mater. Sci..

[9] Sharma Mohit, Bijwe Jayashree (2012). Surface designing of carbon fabric polymer composites with nano and micron sized PTFE particles. J. Mater. Sci..

[10] Akdogan G., Stolarski T.A., Tobe S. (2002). Wear of metal/PTFE coatings in rolling line contact. J. Mater. Sci..

[11] Hubáček T., Lyutakov O., Rybka V., Švorčík V. (2010). Electrical properties of flash-evaporated carbon nanolayers on PTFE. J. Mater. Sci..

[12] DuPont-Fluoroproducts T. (1996). PTFE. Properties Handbook.

[13] Waugh Theodore R. (1972). Polytetrafluoroethylene (Teflon) tools for total arthroplasty of the hip. Clin. Orthop. Relat. Res..

[14] Defrere J., Franckart A. (1992). Teflon/polyurethane arthroplasty of the knee: the first 2 years preliminary clinical experience in a new concept of artificial resurfacing of full thickness cartilage lesions of the knee. Acta Chir. Belg..

[15] Faughnan P., Bryan C., Gan Y., Aglan H. (1998). Correlation between the dynamic mechanical properties and the fatigue behavior of filled and unfilled PTFE materials. J. Mater. Sci. Lett..

[16] Yang S.B., Pu X.X., Huang Z.Y., Wang Q.Y. (2014). Crystalline phase transformation of polytetrafluoroethylene in a fatigue test. J. Appl. Polym. Sci..

[17] Aglan H. (1999). Evaluation of the fatigue fracture resistance of unfilled and filled polytetrafluoroethylene materials. J. Mater. Sci..

[18] Rae P.J., Brown E.N. (2005). The properties of poly (tetrafluoroethylene)(PTFE) in tension. Polymer.

[19] Rae P.J., Dattelbaum D.M. (2004). The properties of poly (tetrafluoroethylene)(PTFE) in compression. Polymer.

[20] Zobaer Shah Quazi Md, Chowdhury Mohammad Asaduzzaman, Kowser Md Arefin (2019). Failure mechanism of polytetrafluoroethylene under friction fatigue. J. Fail. Anal. Prev..

[21] Hobbs J.W. (2000). The effect of eccentric loading on the fatigue performance of high-tensile bolts. Int. J. Fatig..

[22] Meyer R.R. (1967). Buckling of 45° eccentric-stiffened waffle cylinders. Aeronaut. J..

[23] Zobaer Shah Quazi Md, Kowser Md Arefin, Chowdhury Mohammad Asaduzzzaman (2019). A parametric investigation on the fretting fatigue behaviour of heat treated Al 6061-T6 under rotating bending phenomena. Aust. J. Mech. Eng..

[24] Dowson D. (2008). Hip replacement: tribological principles, materials and engineering. Joint Replacement Technology.

[25] Flitney Bob (2005). Composite seal material to compete with PTFE. Seal. Technol..

[26] Ding Jun (2014). Finite element analysis on bending fretting fatigue of 316L stainless steel considering ratchetting and cyclic hardening. Int. J. Mech. Sci..

[27] Blanchet T.A., Kennedy F.E. (1992). Sliding wear mechanism of polytetrafluoroethylene (PTFE) and PTFE composites. Wear.

[28] Zobaer Shah Q.M., Kowser M.A., Chowdhury M.A. (2020). Investigation of the Combined Effect of Notch and Fretting on Bending Fatigue. Theor. Appl. Mech..

